# The causal relationship between COVID-19 and seventeen common digestive diseases: a two-sample, multivariable Mendelian randomization study

**DOI:** 10.1186/s40246-023-00536-x

**Published:** 2023-09-26

**Authors:** Zhiqi Wang, Huanyu Zhou, Shurui Zhang, Fei Wang, Haishan Huang

**Affiliations:** 1 Jiangnan University Affiliated Wuxi Fifth People’s Hospital, Wuxi, 214000 Jiangsu China; 2 The Affiliated Suqian Hospital of Xuzhou Medical University, Suqian, 223800 Jiangsu China; 3 Jiangnan University Affiliated Wuxi Second People’s Hospital, Wuxi, 214000 Jiangsu China; 4 The Shangyou People’s Hospital, Ganzhou, 341000 Jiangxi China

**Keywords:** COVID-19, Mendelian randomization, Common digestive diseases, Gastroesophageal reflux disease

## Abstract

**Objectives:**

In clinical practice, digestive symptoms such as nausea, vomiting are frequently observed in COVID-19 patients. However, the causal relationship between COVID-19 and digestive diseases remains unclear.

**Methods:**

We extracted single nucleotide polymorphisms associated with the severity of COVID-19 from summary data of genome-wide association studies. Summary statistics of common digestive diseases were primarily obtained from the UK Biobank study and the FinnGen study. Two-sample Mendelian randomization analyses were then conducted using the inverse variance-weighted (IVW), Mendelian randomization-Egger regression (MR Egger), weighted median estimation, weighted mode, and simple mode methods. IVW served as the primary analysis method, and Multivariable Mendelian randomization analysis was employed to explore the mediating effect of body mass index (BMI) and type 2 diabetes.

**Results:**

MR analysis showed that a causal association between SARS-CoV-2 infection (OR = 1.09, 95% CI 1.01–1.18, *P* = 0.03), severe COVID-19 (OR = 1.02, 95% CI 1.00–1.04, *P* = 0.02), and COVID-19 hospitalization (OR = 1.04, 95% CI 1.01–1.06, *P* = 0.01) with gastroesophageal reflux disease (GERD). Mediation analysis indicated that body mass index (BMI) served as the primary mediating variable in the causal relationship between SARS-CoV-2 infection and GERD, with BMI mediating 36% (95% CI 20–53%) of the effect.

**Conclusions:**

We found a causal relationship between SARS-CoV-2 infection and gastroesophageal reflux disease. Furthermore, we found that the causal relationship between SARS-CoV-2 infection and GERD is mainly mediated by BMI.

**Supplementary Information:**

The online version contains supplementary material available at 10.1186/s40246-023-00536-x.

## Introduction

COVID-19, caused by the highly infectious and rapidly spreading novel coronavirus SARS-CoV-2, has significantly impacted global public health systems and economic development [1]. The main clinical manifestations of COVID-19 include fever, often with a body temperature above 37.3 °C, which is the initial symptom in most cases. CT imaging reveals multiple small patchy shadows and interstitial changes in the lungs, which can progress to bilateral multiple ground-glass shadows, infiltrative shadows, and even pulmonary consolidation in severe cases [2]. COVID-19 primarily damages the respiratory system, but it can also manifest with digestive symptoms including nausea, vomiting, diarrhea, abdominal pain, and anorexia. A retrospective study [3] found that among 2800 COVID-19 patients, the incidence of digestive symptoms was as follows: diarrhea (7.5%), nausea (4.5%), anorexia (4.4%), vomiting (1.3%), abdominal pain (0.5%), and belching (0.3%). Furthermore, certain studies [4] have indicated that digestive symptoms may be the primary manifestation in some cases, suggesting that they represent a significant manifestation of COVID-19 alongside respiratory symptoms. While numerous studies have indicated a correlation between COVID-19 and digestive diseases, existing observational studies may have biased conclusions due to the presence of confounding factors. To mitigate the impact of confounding factors on the relationship between COVID-19 and digestive diseases, a more effective method is required to infer potential causal relationships.

Mendelian randomization (MR) can mitigate the impact of confounding factors. Its fundamental principle is that genetic variations are randomly assigned at conception, and a trait is typically uncorrelated with other traits. This process is akin to randomly assigning participants to treatment and control groups in a randomized controlled trial [5]. MR design also minimizes reverse causality as alleles are fixed at birth and cannot change with the occurrence or development of diseases. Utilizing genetic variation as an instrumental variable to infer causal relationships between exposure and outcome [6] can mitigate the interference of confounding factors [7]. MR can overcome certain limitations of observational studies (confounding, reverse causality, regression dilution bias) and randomized controlled trials (representativeness and feasibility issues) in causal inference [8]. With the identification of numerous genetic variations closely associated with specific traits in the field of biology, and the availability of summary data on the relationship between exposure, disease, and genetic variation from numerous large-scale genome-wide association studies (GWAS), researchers can estimate genetic associations using extensive datasets. In this study, we employ the two-sample and multivariable MR methods to examine the causal relationship between COVID-19 and common digestive diseases, while exploring the potential mediating effects of underlying factors on this relationship.

## Materials and methods

### Design

**Fig. 1:**
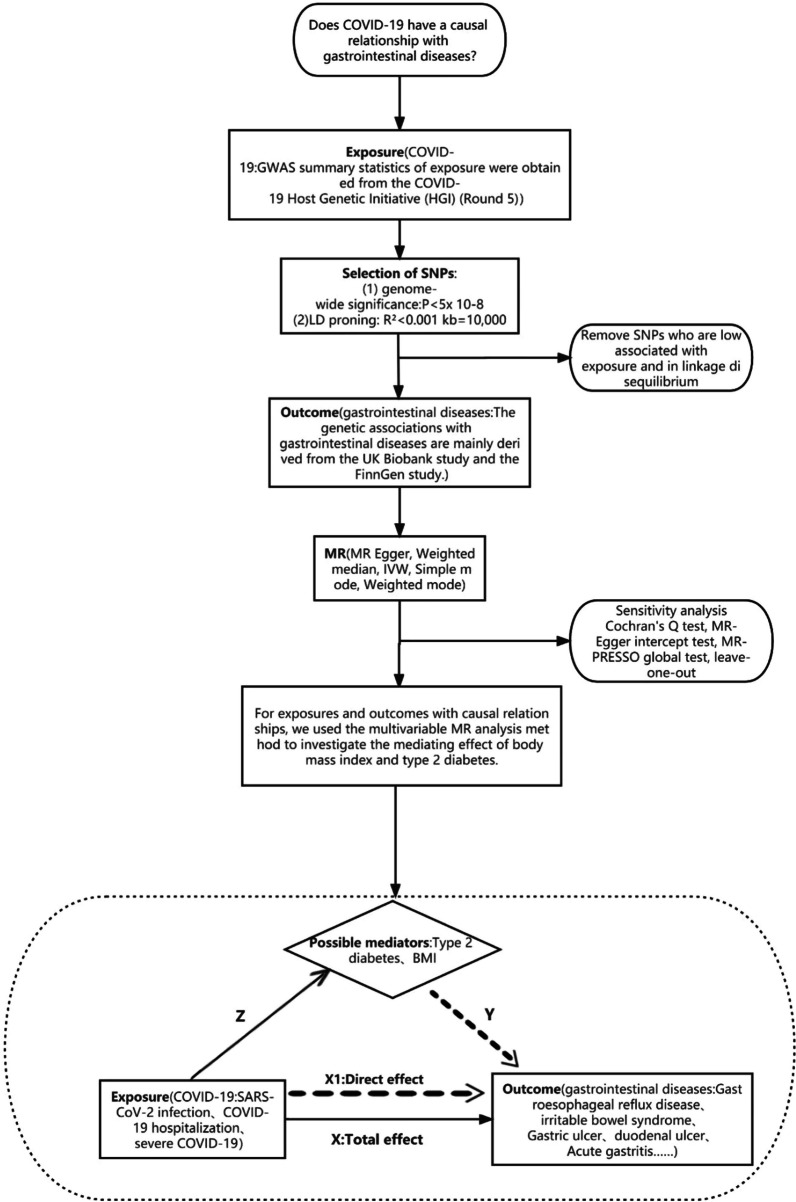
This figure is a flow chart of the article. ① Z: effects of exposure on possible mediators, X: effects of exposure on outcome, X1: effect of exposure on the outcome after adjusting for potential mediators, Y: effect of potential mediators on the outcome after adjusting for exposure; ② MR satisfies the following three assumptions: (1) there is a strong association between the instrumental variable A and the exposure factor B; (2) the instrumental variable A is not associated with any confounding factors D that are related to the association between A and B–C; (3) the instrumental variable A does not affect the outcome C unless it is possibly achieved through its association with the exposure B

We extracted eligible instrumental variables (IVs) from GWAS summary data on COVID-19 and 17 common digestive diseases. SNPs significantly associated with COVID-19 were used as the IVs. The outcome variable was the presence of the 17 common digestive diseases, and a TSMR analysis was conducted. For exposures and outcomes with causal relationships, we further examined the causal relationship between potential mediators and outcomes, as well as the causal relationship between exposures and potential mediators. Once the true mediators were identified, we employed the Multivariable Mendelian randomization (MVMR) analysis to estimate the effect of these mediators on the outcome. We then multiplied this effect by the effect of exposure on the mediators to determine the mediator effect. Finally, we divided the mediator effect by the total effect of exposure on the outcome to calculate the proportion of mediation effect for each mediator (Fig. 1).

### Data sources

The GWAS data for COVID-19 were obtained from the COVID-19 Host Genetic Initiative (HGI) (Round 5), encompassing three categories: SARS-CoV-2 infection (cases: controls = 38,984:1,644,784), COVID-19 hospitalization (cases:controls = 9986:1,877,672), and severe COVID-19 (cases:control = 5101:1,383,241). The GWAS data for 17 common digestive diseases were primarily derived from the UK Biobank study and the FinnGen study, including Gastroesophageal reflux disease (cases:controls = 129,080:473,524), irritable bowel syndrome (cases:controls = 10,939:451,994), Gastric ulcer (cases:controls = 1834:35,936), duodenal ulcer (cases:controls = 1908:461,025), Acute gastritis (cases:controls = 1284:189,695), Chronic gastritis (cases:controls = 5213:189,695), Ulcerative colitis (cases:controls = 4320:210,300), Nonalcoholic fatty liver disease (cases:controls = 894:217,898), Cholangitis (cases:controls = 778:195,144), Cholelithiasis (cases:controls = 19,023:195,144), Chlocystitis (cases:controls = 2013:195,144), Acute pancreatitis (cases:controls = 3022:195,144), Chronic pancreatitis (cases:controls = 1737:195,144), Acute appendicitis (cases:controls = 11,899:201,886), Crohn’s disease (cases:controls = 732:336,467), Colorectal cancer (cases:controls = 5657:372,016), and Gastric cancer (cases:controls = 6563:195,745). Based on previous observational and MR studies, we considered BMI and type 2 diabetes [9, 10] as potential mediators. The BMI data (Sample size: Number of SNPs = 336,107:10,894,596) were obtained from the UK Biobank study, while the type 2 diabetes data (cases:controls = 74,124:824,006) were obtained from the latest meta-analysis by Mahajan et al. based on two European ancestry GWASs. The populations in the aforementioned data sources, except for gastric cancer (East Asian ancestry), consisted of individuals of European ancestry, including both males and females. All datasets used in this study are publicly available, and ethical approval and written informed consent were obtained in the original studies (Additional file 1).

### Selection of instrumental variables

We collected SNPs significantly associated with COVID-19 (*P* < 5 × 10^−8^) and removed SNPs in strong linkage disequilibrium (LD) (r^2^ < 0.001, 10,000 kb) to avoid biased results [11]. SNPs associated with digestive diseases (*P* < 5 × 10^−8^) were excluded. To ensure a strong correlation with the exposure, we selected SNPs with an F statistic > 10 as IVs [12]. The F statistic was calculated using the formula F = R^2^(N − K − 1)/K(1 − R^2^), where R^2^ was calculated using the formula R^2^ = (2 × EAF × (1 − EAF) × Beta^2^)/[(2 × EAF × (1 − EAF) × Beta^2^) + (2 × EAF × (1 − EAF) × N × SE^2^)] [13, 14]. Palindromic SNPs with intermediate allele frequencies (allele frequencies between 0.01 and 0.30) were also removed [15]. Palindromic SNPs refer to SNPs where the alleles correspond to nucleotides that are paired with each other in a DNA molecule. In cases where SNP data were unavailable in the GWAS, proxy SNPs were obtained using the LDlink online platform (https://ldlink.nci.nih.gov/).

### Statistical analysis

In this study, we primarily employed the IVW, MR-Egger regression, and weighted median methods for MR analysis. The IVW method involves weighting each instrumental variable by the inverse of its variance, assuming the absence of an intercept, and calculating the weighted average of the effect estimates from all instrumental variables. The MR-Egger method differs from IVW by considering the presence of an intercept during regression and using the inverse of the outcome variance as a weight for fitting. The Weighted Median Estimation (WME) is defined as the median of the weighted empirical density function of the ratio estimate. If at least half of the instruments are valid, the causal relationship can be consistently estimated. The heterogeneity test examines the differences between instrumental variables, where larger differences indicate greater heterogeneity. A random-effects model was used in this study to estimate the MR effect size. The pleiotropy test assesses whether multiple instrumental variables exhibit horizontal pleiotropy, commonly indicated by the intercept term in MR-Egger regression. A significant deviation of the intercept term from zero suggests the presence of horizontal pleiotropy [16]. The leave-one-out sensitivity test calculates the MR result with one instrumental variable excluded at a time [17]. If the MR result with the remaining instrumental variables significantly differs from the overall result after excluding a specific instrumental variable, it indicates the sensitivity of the MR result to that variable. Additionally, to validate the robustness of the results, MR pleiotropy residual sum and outlier (MR-PRESSO) were used to detect outliers. If outliers were identified, they were removed, and the analysis was repeated. All statistical analyses were conducted using R version 4.2.1 (R Foundation for Statistical Computing, Vienna, Austria). The Two-sample Mendelian randomization (TSMR) (version 0.5.6) [12] and MR-PRESSO (version 1.0) [18] R packages were employed for the MR analysis.

## Results

### Selection of SNPs

Based on the significant association with COVID-19 (*P* < 5 × 10^−8^) and the removal of LD between SNPs (r^2^ < 0.001, 10,000 kb), we selected 7 SNPs (rs10936744, rs12482060, rs17078348, rs2271616, rs4971066, rs643434, rs757405) for SARS-CoV-2 infection, 7 SNPs (rs10860891, rs111837807, rs13050728, rs2109069, rs2384074, rs35081325, rs77534576) for severe COVID-19, and 5 SNPs (rs13050728, rs2109069, rs2660, rs35081325, rs505922) for COVID-19 hospitalization in subsequent research. The F values for all instrumental variables exceeded 10, indicating a low likelihood of causal association being influenced by weak instrumental variable bias.

#### Two-sample Mendelian randomization

**Fig. 2:**
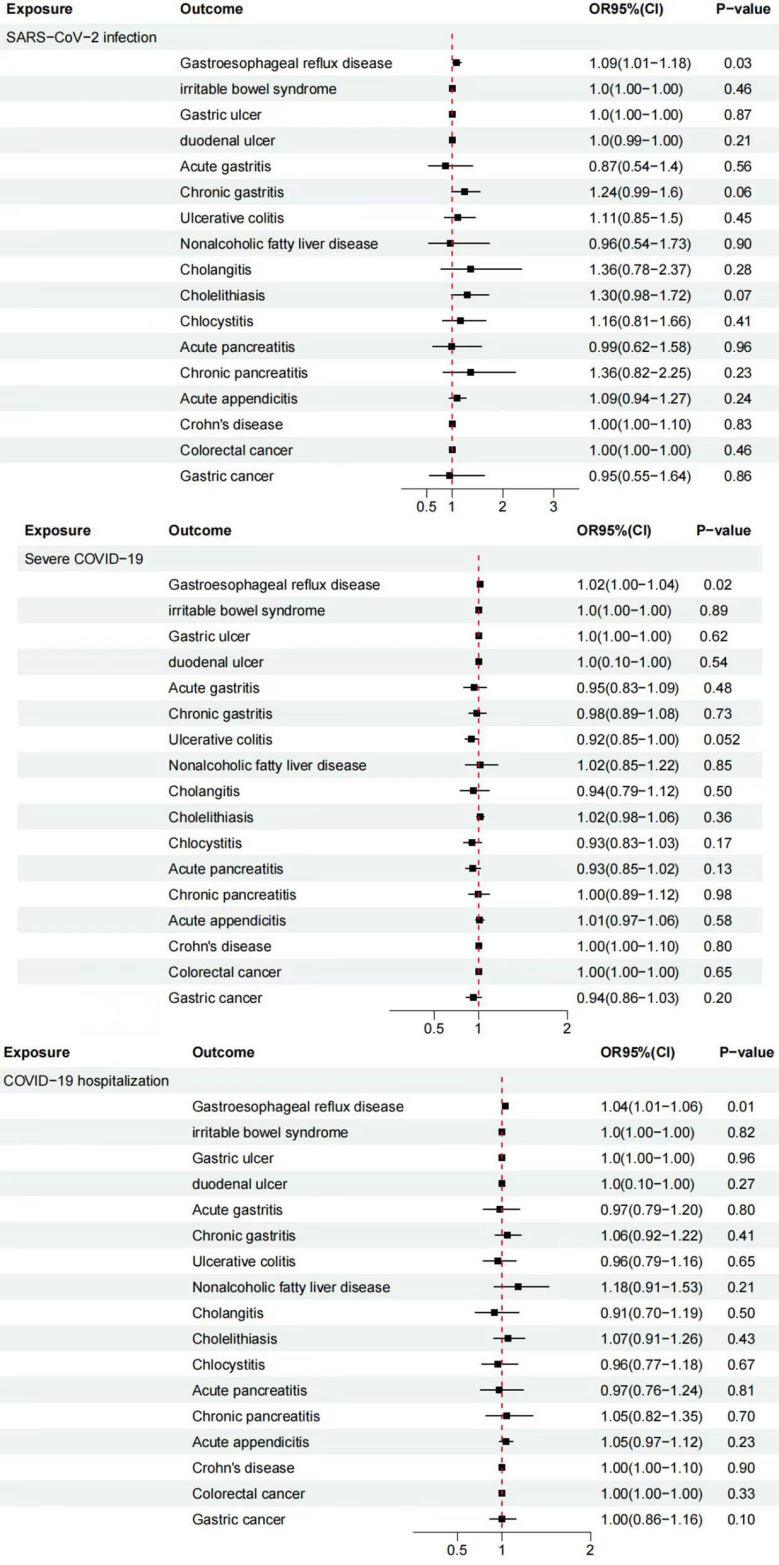
TSMR estimates for the association between COVID-19 and gastrointestinal diseases

In the MR analysis of 17 common digestive diseases using the IVW method, it was found that SARS-CoV-2 infection (OR = 1.09, 95% CI 1.01–1.18, *P* = 0.03), severe COVID-19 (OR = 1.02, 95% CI 1.00–1.04, *P* = 0.02), and COVID-19 hospitalization (OR = 1.04, 95% CI 1.01–1.06, *P* = 0.01) was causally associated with GERD and may increase the risk of developing it. Sensitivity analysis revealed no heterogeneity among SNPs in the Cochran Q tests for IVW (SARS-CoV-2 infection: *P* = 0.62, severe COVID-19: *P* = 0.54, COVID-19 hospitalization: *P* = 0.50) and MR-Egger regression (SARS-CoV-2 infection: *P* = 0.41, severe COVID-19: *P* = 0.41, COVID-19 hospitalization: *P* = 0.34). Additionally, the difference between the egger_intercept and 0 in MR-Egger was not statistically significant for SARS-CoV-2 infection (*P* = 0.92), severe COVID-19 (*P* = 0.66), and COVID-19 hospitalization (*P* = 0.68), indicating no presence of horizontal pleiotropy among SNPs. Furthermore, the “Leave-one-out” sensitivity analysis showed that the IVW analysis results for the remaining SNPs were similar to the analysis results for all SNPs included, and no SNPs significantly affected the estimated causal associations. The MR-PRESSO analysis results also indicated that there were no significant outliers among the instrument variable sites included in the study that affected the results (Fig. 2, Additional file 2).

#### Multivariable Mendelian randomization

**Fig. 3:**
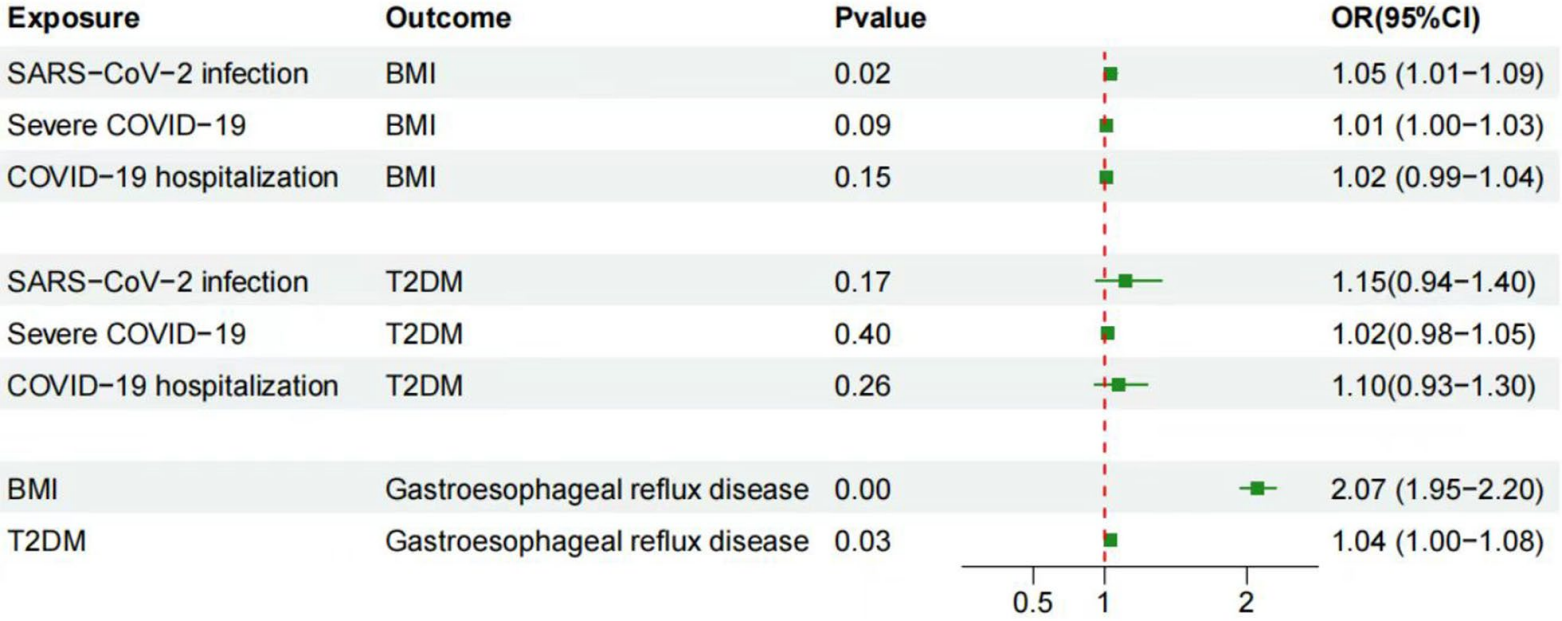
Using the TSMR method to find mediators

**Fig. 4:**
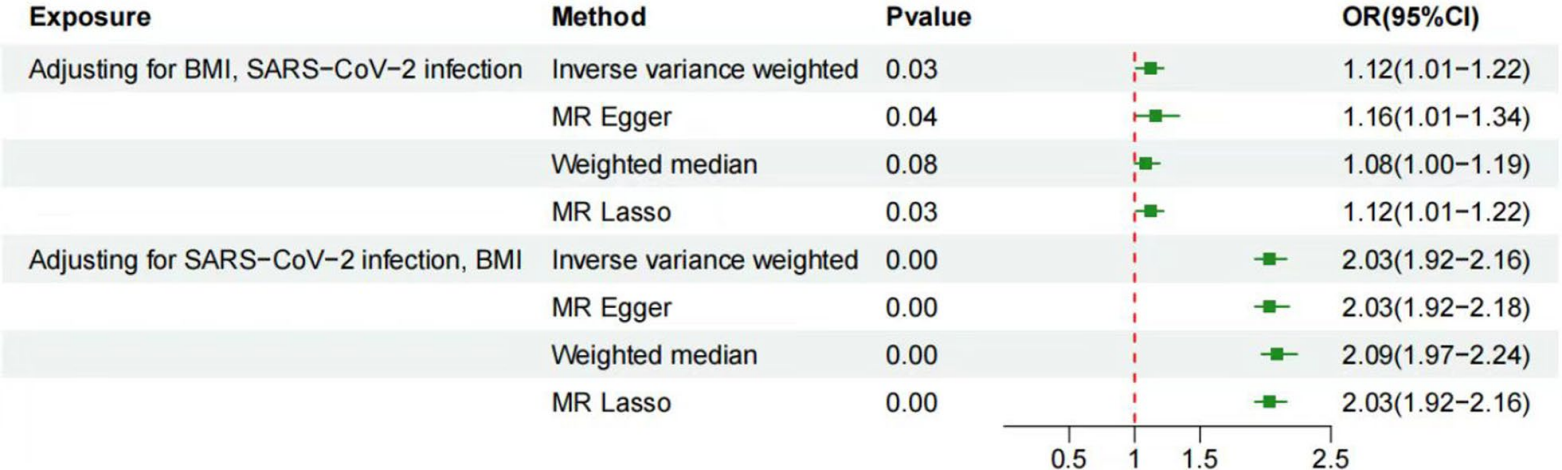
Evaluate the effect of exposure on the outcome after adjusting for the mediator using the MVMR method

Firstly, we conducted TSMR analyses with BMI and type 2 diabetes as exposures and GERD as the outcome. The IVW method showed that BMI (OR = 2.07, 95% CI 1.95–2.20, *P* = 8.7E−127) and type 2 diabetes (OR = 1.04, 95% CI 1.00–1.08, *P* = 0.03) were causally associated with an increased risk of GERD. Next, we selected COVID-19 as the exposure and BMI and type 2 diabetes as the outcomes. The IVW method revealed that only SARS-CoV-2 infection (OR = 1.05, 95% CI 1.01–1.09, *P* = 0.02) was causally related to BMI. Finally, we performed MVMR analysis with SARS-CoV-2 infection and BMI as exposures and GERD as the outcome. The IVW method demonstrated that SARS-CoV-2 infection after adjusting for BMI (OR = 1.11, 95% CI 1.01–1.23, *P* = 0.03) and BMI after adjusting for SARS-CoV-2 infection (OR = 2.03, 95% CI 1.91–2.17, *P* = 1.65E−109) were causally related to GERD. The sensitivity analysis identified heterogeneity among SNPs when used as instrumental variables, as indicated by the Cochran Q test (*P* < 0.01), suggesting potential pleiotropy of SNP effects. The MR-PRESSO method also detected heterogeneity (*P* < 0.01) but did not identify any outliers. To further investigate potential pleiotropy and invalid SNPs, we conducted MR-Egger regression and MR-Lasso methods. The results of MR-Egger regression showed that the difference between the Egger intercept and zero was not statistically significant (*P* = 0.41), and MR-Lasso did not detect any abnormal SNPs. Therefore, we concluded that there was no evidence of directional pleiotropy in this case (*P* > 0.05) [19]. Furthermore, the MR-weighted median method showed consistent results with MR-IVW in both magnitude and direction, indicating that horizontal pleiotropic effects did not significantly bias our results (Fig. 3, 4, Additional files 3, 4, 5, 6, 7). According to the above analysis, we obtained a total effect size of 0.087 for the association between SARS-CoV-2 infection and GERD, and a mediation effect size of 0.031 for BMI. Finally, to evaluate the presence of a reverse causal relationship between COVID-19 and GERD, as well as to gain a deeper understanding of the causal association between the two, we conducted a bidirectional Mendelian randomization analysis. The SNP selection criteria remained the same as before. After controlling for confounding factors, we conducted Mendelian randomization analysis using GERD as the exposure and COVID-19 (SARS-CoV-2 infection) as the outcome. The IVW results showed no causal association between GERD (OR = 1.15, 95% CI 0.98–1.33, *P* = 0.08) and SARS-CoV-2 infection. Sensitivity analysis revealed no heterogeneity or horizontal pleiotropy, confirming the reliability of these results. This further supports the positive causal relationship between SARS-CoV-2 infection and GERD (Additional file 9).

## Discussion

We identified a causal relationship between SARS-CoV-2 infection and GERD, with BMI as an important mediating factor, through a Mendelian randomization study investigating the associations between three exposures to COVID-19 (SARS-CoV-2 infection, severe COVID-19, and COVID-19 hospitalization) and 17 common gastrointestinal diseases. This finding may explain why COVID-19 patients often experience gastrointestinal symptoms in clinical practice. Interventions targeting these mediating factors may alleviate such symptoms in COVID-19 patients.

In the early stages of the COVID-19 pandemic, clinical manifestations of the disease outside the respiratory system were often overlooked due to a lack of understanding and clinical observation. However, clinical observations now suggest that over half of COVID-19 patients experience gastrointestinal symptoms, such as anorexia, diarrhea, nausea, vomiting, and abdominal pain. The specific mechanisms behind these symptoms remain unclear, but some researchers believe that the gastrointestinal tract may be a potential route of transmission and a target organ for SARS-CoV-2. A meta-analysis of 4243 patients revealed that 70.3% of patients’ fecal samples were positive for SARS-CoV-2 nucleic acid, even after respiratory SARS-CoV-2 nucleic acid testing was negative. Moreover, the viral load in the feces of patients with diarrhea was higher than that of patients without diarrhea [20]. Another study detected SARS-CoV-2 nucleic acid in fecal samples from hospitalized COVID-19 patients, including those with and without gastrointestinal symptoms [21]. Some scholars have suggested that the angiotensin-converting enzyme 2 (ACE2), which is widely present in the gastrointestinal tract, may be related to gastrointestinal symptoms in COVID-19 patients [22]. ACE2 is the host receptor for SARS-CoV-2 infection in human cells, and the spike protein on the surface of SARS-CoV-2 can bind to the ACE2 receptor, causing cell damage [22]. The receptor-binding domain (RBD) of the spike protein has a high similarity to that of SARS-CoV-2, resulting in a strong affinity between SARS-CoV-2 and ACE2 [23]. SARS-CoV-2 can invade the gastrointestinal tract by utilizing common receptors or auxiliary proteins as partners of ACE2, leading to a series of gastrointestinal symptoms [24].

GERD is caused by the reflux of gastric and duodenal contents into the esophagus, resulting in esophageal symptoms and complications. Previous studies have identified BMI as a significant risk factor for GERD [25]. Our study confirms these findings, and other major risk factors include pregnancy, smoking, alcohol consumption, chocolate, fatty foods, anatomical defects (such as hiatal hernia), and the use of certain medications such as aspirin, antihistamines, and calcium channel blockers [26]. Our study also establishes a causal relationship between SARS-CoV-2 infection and GERD, which is consistent with some previous MR studies. In a 2021 MR study, Ong [27] et al. discovered a small to moderate genetic correlation between GERD and COVID-19. The OR for the severity of COVID-19 was 1.15 (95% CI 0.96–1.39), while the OR for the risk of COVID-19 hospitalization was 1.16 (1.01–1.34). After adjusting for known COVID-19 risk factors (BMI, smoking, type 2 diabetes, and coronary artery disease), the genetic correlation between GERD and COVID-19 was slightly weakened, indicating that BMI is an important risk factor for both diseases. Our study, which had a larger sample size and investigated exposure and outcomes, not only replicated Ong’s findings but also demonstrated the causal relationship between the two diseases in reverse. We also found that BMI is a significant mediating factor between the two diseases, and interventions aimed at reducing BMI may decrease the risk of COVID-19 patients developing GERD.

BMI is widely recognized as a characteristic of obesity, and several studies have established its association with COVID-19 [28]. A study analyzed data from COVID-19 patients in the American Heart Association, grading their BMI according to the World Health Organization (WHO) obesity classification. Among 7606 patients under 50 years of age with a high BMI (≥ 25.0 kg/m^2^), the risk of in-hospital death and mechanical ventilation increased with increasing BMI values (Additional file 8; Fig. 1) [29]. Another MR study found that the association between BMI and COVID-19 outcomes persisted even after adjusting for waist circumference, trunk fat ratio, cardiovascular disease, and type 2 diabetes [30]. BMI also plays a significant mediating role in the causal relationship between COVID-19 and other diseases, such as heart disease [31]. Some researchers [32] suggest that the increase in BMI leading to severe COVID-19 may be related to increased macrophage infiltration, abnormal secretion of pro-inflammatory cytokines and insulin, leading to systemic immune dysfunction. Our study’s results, which are consistent with previous research, demonstrate that BMI has a significant mediating effect of up to 36% on the causal relationship between SARS-CoV-2 infection and GERD. This finding provides valuable insights for managing the gastrointestinal symptoms of COVID-19 patients in clinical practice.

Although we have confirmed the causal relationship between COVID-19 and GERD, we cannot rule out the possibility of a relationship between COVID-19 and the other 16 gastrointestinal diseases due to various factors, such as inadequate sample size, genotype selection bias, or unknown factors. Therefore, further research in a larger population is needed to verify these results and determine the relationship between COVID-19 and other gastrointestinal diseases.

Our MR study effectively avoided confounding factors and saved time and resources compared to observational studies. We conducted multivariable MR studies to verify the results and explore potential mediators. However, our study has certain limitations, including limited data on individuals of non-European descent, which may affect the generalizability of our findings. Additionally, as with all MR studies, unobserved pleiotropy cannot be completely ruled out, which may introduce bias into our results. Furthermore, clinical symptoms of GERD patients may be influenced by short-term factors, such as food intake and emotions, which limit the applicability of our conclusions for guiding clinical treatment.

## Conclusion

Our study employed two-sample MR and multivariable MR methods to investigate the causal relationship between COVID-19 and common gastrointestinal diseases. We found strong evidence for a causal effect of SARS-CoV-2 infection on GERD and further showed that approximately one-third of the excess risk of GERD in COVID-19 patients is mediated by BMI. This underscores the importance of interventions targeting BMI, which may alleviate digestive symptoms in COVID-19 patients.

### Supplementary Information

**Additional file 1.** Exposure, mediation, and outcome data sources.

**Additional file 2.** Mendelian randomization analysis of 17 digestive diseases.

**Additional file 3.** The TSMR analysis of COVID19 and type 2 diabetes.

**Additional file 4.** The TSMR analysis of COVID19 and BMI.

**Additional file 5.** The TSMR analysis of type 2 diabetes and GERD.

**Additional file 6.** The TSMR analysis of BMI and GERD.

**Additional file 7.** The MVMR analysis of COVID19, BMI and GERD.

**Additional file 8: Fig. 1.** The impact of different BMI stratification on the risk of hospital death or mechanical ventilation.

**Additional file 9.** Mendelian randomization analysis of reflux esophagitis and SARS-CoV-2 infection.

## Data Availability

Research data are reasonably available from the authors.

## Abbreviations

SNPs: Single nucleotide polymorphisms
GWAS: Genome-wide association studies
IVW: Inverse variance-weighted
MR Egger: Mendelian randomization-Egger regression
WME: Weighted median estimation
BMI: Body mass index
GERD: Gastroesophageal reflux disease
MR: Mendelian randomization
IVs: Instrumental variables
HGI: Host genetic initiative
LD: Linkage disequilibrium
MR-PRESSO: MR pleiotropy residual sum and outlier
TSMR: Two-sample Mendelian randomization
ACE2: Angiotensin-converting enzyme 2
RBD: Receptor-binding domain
WHO: World Health Organization
MVMR: Multivariable Mendelian randomization
