# Supplementary figures and images for: The causal relationship between COVID-19 and seventeen common digestive diseases: a two-sample, multivariable Mendelian randomization study
Source: Hum Genomics. 2023 Sep 26;17:87. doi: 10.1186/s40246-023-00536-x (PMC10523605; doi:10.1186/s40246-023-00536-x)

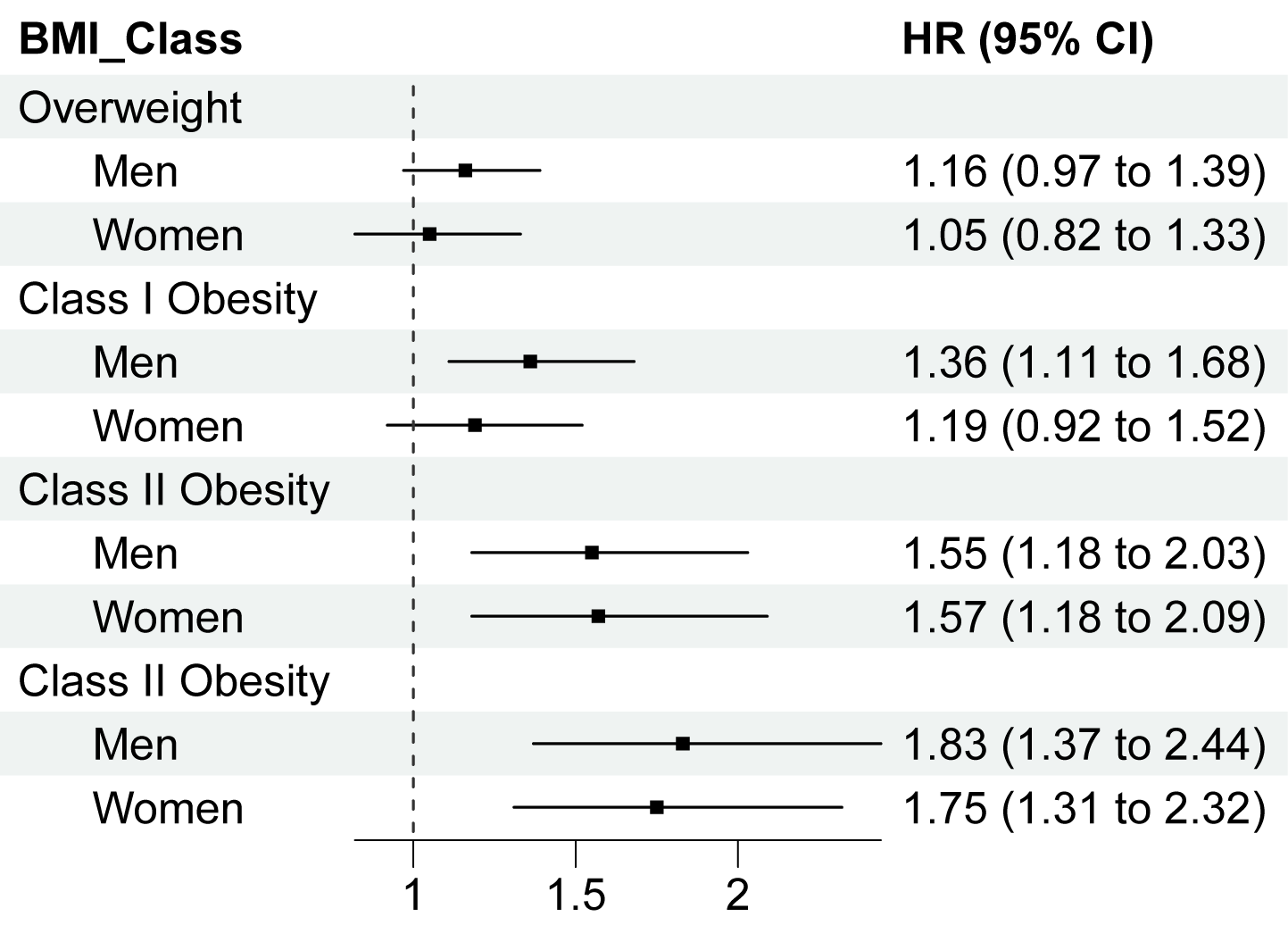

Supplement: Supplementary file 8 — Additional file 8: Fig. 1. The impact of different BMI stratification on the risk of hospital death or mechanical ventilation. [file 40246_2023_536_MOESM8_ESM.tif]
